# Supplementary material for: Ecological Flow Response Analysis to a Typical Strong Hydrological Alteration River in China
Source: Int J Environ Res Public Health. 2023 Jan 31;20(3):2609. doi: 10.3390/ijerph20032609 (PMC9916225; doi:10.3390/ijerph20032609)
Supplement: Supplementary file 1 [file ijerph-20-02609-s001.zip › ijerph-2133434-supplementary.pdf]

Supplementary Materials

1 The calculation steps of GI

The specific steps for constructing the annual distribution uniformity of monthly runoff based on GI are as follows.

1) Divide the monthly runoff data from the same year into one group, and then arrange the monthly runoff data from each group in ascending order.

2) Accumulate the monthly runoff data in accordance with time accumulation (based on month).

3) Plot the Lorenz curve for each group, with the horizontal coordinate representing the cumulative time to total time and the vertical coordinate representing the cumulative monthly runoff to the sum of runoff. The Lorenz curve with 1954 runoff data was drawn as an example (**Supplementary Figure S1**).

4) To calculate GI, using formula (S1).

$$\mathrm{GI} = \frac{S_B}{S_A + S_B} \tag{S1}$$

Where S_A and S_B represent the area of A and B in **Supplementary Figure S1**, respectively.

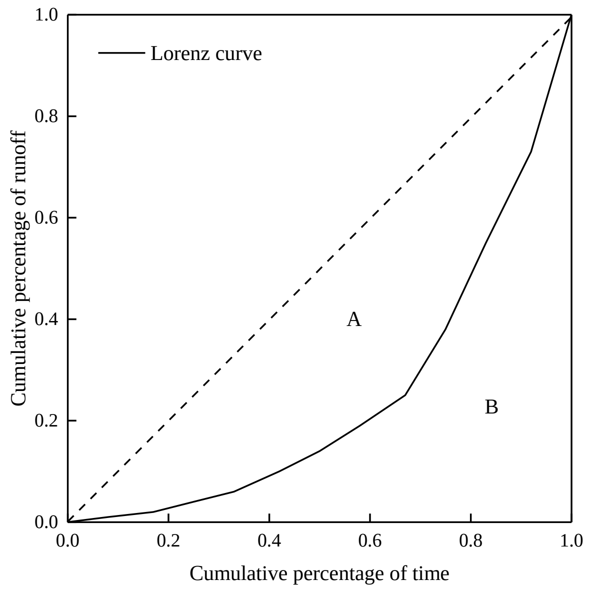

Supplementary Figure S1. Lorenz curve of annual distribution for monthly runoff in 1954 (GI = 0.494).
